# Role of Nanoplastics in Decreasing the Intestinal Microbiome Ratio: A Review of the Scope of Polystyrene

**DOI:** 10.3390/toxics13121036

**Published:** 2025-11-29

**Authors:** Steven C. Sutton, Ronald D. Hills

**Affiliations:** 1 Department of Pharmaceutical Sciences and Administration, School of Pharmacy, Westbrook College of Health Professions, University of New England, Portland, ME 04103, USA; rhills@une.edu; 2 Portland Laboratory for Biotechnology and Health Sciences, University of New England, Portland, ME 04103, USA

**Keywords:** microplastic, nanoplastic, *Bacteroidetes*, *Firmicutes*, Gram-negative, Gram positive, microbiome dysbiosis

## Abstract

Micro- and nanoplastics (MNPs) are increasingly recognized as emerging intestinal toxicants. This scoping review maps and integrates evidence from 56 studies (47 primary and 11 review articles, 2000–mid-2025) on how nanoplastics, particularly ≤100 nm polystyrene, disrupt gut homeostasis. The evidence consistently supports a three-stage mechanistic cascade: 1. Oxidative-stress initiation—Nanoplastics generate reactive oxygen species (ROS) and suppress antioxidant defenses, producing redox imbalance in intestinal tissue and commensal bacteria. 2. Barrier dysfunction—Resulting oxidative injury reduces tight-junction proteins, depletes mucus-secreting goblet cells, and activates inflammatory signaling (NF-κB, TLR4). 3. Microbiome reconfiguration—The altered intestinal microenvironment favors Gram-negative expansion and depletion of Gram-positive commensals, observed as decreases in the *Firmicutes*/*Bacteroidetes* (F/B) and Gram+/Gram− ratios. High-dose nanoplastic exposures reproducibly induced these effects in mice and zebrafish, whereas environmentally realistic, low-dose PET fragments produced minimal dysbiosis. Functionally important taxa—short-chain-fatty-acid producers (*Faecalibacterium*, *Roseburia*) and mucin degraders (*Akkermansia muciniphila*)—were consistently reduced, linking microbial shifts to epithelial injury and inflammatory tone. Together, these findings define an oxidative–barrier–microbiome axis as the dominant pathway of nanoplastic-induced intestinal disruption. Future work should emphasize environmentally relevant exposures, multi-omics functional endpoints, and mechanistic models that integrate oxidative stress, epithelial pathology, and microbiome ecology to guide realistic human-health risk assessment.

## 1. Introduction

### 1.1. Micro- and Nanoplastic Pollution as an Emerging Health Concern

“The Plastic Within and Without You” means plastic is in the environment (water, fish, plants, soil animals, birds) and in the body (feces, GI tissue, liver, blood, brain, placenta, testes), with infant exposure even more so than adults [1]. Microplastics (MPs, <5 mm) have been widely detected in food, water, and air, with human ingestion now well documented [2]. Increasing attention is now directed toward nanoplastics, which display greater biological reactivity due to their small size, surface charge, and ability to cross epithelial and cellular barriers. Nanoplastics are most strictly defined as plastic particles < 100 nm, consistent with nanoparticle toxicology. However, some reviews and agencies adopt a broader cutoff of <1000 nm. In this review, we define nanoplastics operationally as ≤100 nm, in line with recent in vivo gut microbiome studies. The gastrointestinal tract is therefore a primary interface where particle exposure may disrupt host systems.

### 1.2. Gut Microbiome as a Target of Disruption

The gut microbiome plays a central role in host energy metabolism, immune regulation, and barrier function [3]. Alterations in its composition—often termed dysbiosis—have been linked to obesity, diabetes, inflammatory bowel disease, and other chronic disorders. Common summary metrics include the *Firmicutes*/*Bacteroidetes* (F/B) ratio and measures of alpha diversity, but both have limitations in sensitivity and reproducibility [4]. Increasingly, attention has shifted to functionally important groups such as short-chain fatty acid (SCFA) producers (*Faecalibacterium*, *Roseburia*), mucin degraders (*Akkermansia muciniphila*), and bile salt hydrolase (BSH)-rich taxa, which provide more mechanistic insights into host–microbe interactions [5].

### 1.3. Previous Reviews and Current Gaps

Several reviews have examined plastics and the gut microbiome (Table 1). These include broad surveys of microplastic exposure and biodegradation, targeted assessments of endocrine disruption, and recent syntheses linking MNP exposure to intestinal inflammation and barrier dysfunction. However, most do not clearly distinguish between MPs and NPs, despite major differences in particle size, ability to enter cells, and mechanisms of reactive oxygen species (ROS) induction. Importantly, few reviews extract microbial taxa-level outcomes or host endpoints in a standardized way, limiting cross-study comparability.

### 1.4. Aim of This Scoping Review

To address these gaps, this scoping review systematically charts in vivo oral-gavage nanoplastic studies (≤100 nm) published between 2020 and mid-2025. Using a structured extraction form, we compiled study descriptors (host species, particle type and surface modification, dose, and exposure duration), microbiome outcomes (*Firmicutes*/*Bacteroidetes* ratio, diversity indices, and taxa-level shifts), and host endpoints (epithelial, immune, oxidative-stress, and systemic responses). To improve comparability, we applied a Gram-positive/Gram-negative weighting approach and critically evaluated methodological quality and integrity risks. Because most animal studies use nominal doses in the tens to hundreds of mg kg^−1^ day^−1^ range—four to five orders of magnitude above modeled human dietary intakes (~µg kg^−1^ day^−1^)—our synthesis explicitly differentiates high-dose mechanistic evidence from environmentally relevant exposure levels. By situating nanoplastic-induced microbiome changes within both toxicological and ecological frameworks, this review clarifies reproducible patterns of disruption and proposes a standardized, dose-contextualized framework for future experimental design.

## 2. The Intestinal Microbiome

**Figure 1:**
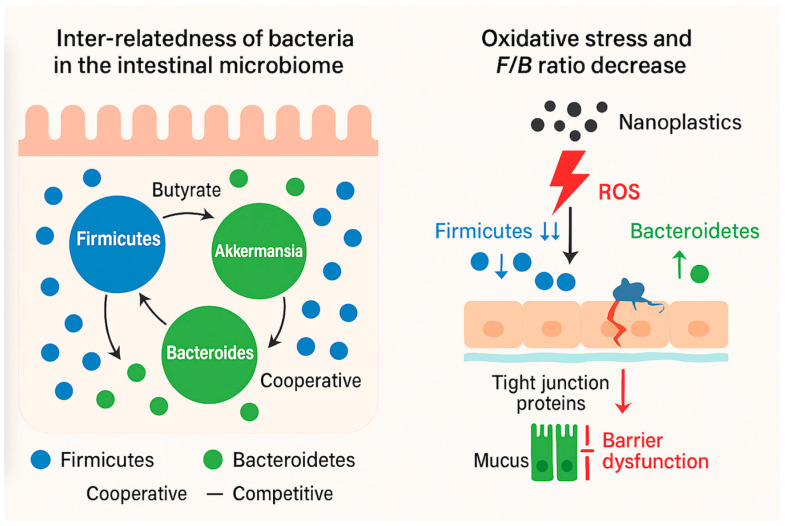
Principles shaping intestinal microbiome ecology and vulnerability to nanoplastic disruption.

The intestinal microbiome is a highly dynamic, interconnected ecosystem composed of trillions of microorganisms that tightly interface with host physiology [17]. These microbes influence one another through cooperation and competition, creating cascading effects on community structure and host health [18]. Before addressing how MNPs perturb these systems, we outline four principles that shape gut ecology: microbial inter-relatedness, niche structuring, keystone species and community stability, and ecological succession and resilience (Figure 1).

The left panel illustrates the inter-relatedness of gut bacteria through metabolic cross-feeding, competition, and cooperative interactions that maintain community stability. Keystone taxa such as *Faecalibacterium prausnitzii*, *Bacteroides thetaiotaomicron*, and *Akkermansia muciniphila* support barrier function, immune regulation, and metabolic diversity. The right panel highlights how oxidative stress, a common response to nanoplastic exposure, disrupts this ecological balance by damaging the intestinal barrier and favoring Gram-negative expansion. This shift is often reflected in a decreased *Firmicutes*/*Bacteroidetes* (F/B) ratio, a reproducible marker of dysbiosis in nanoplastic studies.

### 2.1. Microbiome Bacteria Inter-Relatedness

Gut bacteria interact through ecological and metabolic dependencies that collectively maintain homeostasis and influence disease risk [18]. Primary degraders (e.g., *Bacteroides* spp.) break down complex polysaccharides, releasing intermediates that secondary fermenters convert to short-chain fatty acids (SCFAs)—butyrate, propionate, and acetate—key metabolites for colonocyte energy and immune regulation [19,20]. *Faecalibacterium prausnitzii* consumes acetate to generate butyrate, a canonical cross-feeding example linked to barrier support and anti-inflammatory tone [20].

Competitive Interactions. Microbes compete for nutrients and niches and deploy antagonistic factors such as bacteriocins, microcins, and organic acids to inhibit rivals [21]. Many *Lactobacillus* spp. acidify their micro-environment and contribute to competitive exclusion of pathogens [22].

Cooperative Interactions. Co-aggregation and biofilm formation can protect participants and coordinate function [23]. Quorum-sensing signals mediate interspecies communication and group behaviors within gut consortia [24].

### 2.2. Niche Structuring

By calming immune responses, *B. fragilis* creates a less hostile mucosal environment and allows for colonization by other commensals that are sensitive to inflammation. This helps structure a stable, low-inflammation microbial niche that favors beneficial anaerobes over opportunistic pathogens. In contrast, inflammation (e.g., from infections or poor diet) favors facultative anaerobes like *Enterobacteriaceae*, which thrive in oxygen-rich, inflamed environments [18,25]. Thus, immune modulation by commensals like *B. fragilis* indirectly prevents this pathogenic shift, helping maintain microbial balance [26,27]. This example illustrates how specific microbes do not merely live in the gut but actively shape the ecological and immunological landscape, influencing which other microbes can survive and thrive [17,28].

To avoid excessive attribution of *Firmicutes*/*Bacteroidetes* (F/B) shifts solely to micro- and nanoplastics, we note that several external regulatory factors are also well-established modulators of this ratio. High-fat diets, antibiotic exposure, and host inflammatory states each reshape gut community structure, often producing F/B changes that resemble those observed under nanoplastic exposure. By foregrounding these influences under a dedicated heading, we situate plastic-induced dysbiosis within the broader ecological landscape of diet, pharmacologic disturbance, and inflammation, underscoring that functional taxa-level changes may provide stronger mechanistic signals than the F/B ratio alone.

### 2.3. Keystone Species and Community Stability

Keystone taxa exert outsized functional effects. The butyrate-producer *F. prausnitzii* supports epithelial integrity and dampens inflammation; its depletion is repeatedly linked to Crohn’s disease and dysbiosis [28]. *Bacteroides thetaiotaomicron* carries extensive polysaccharide utilization loci (PULs) that initiate complex carbohydrate digestion and seed trophic chains used by other microbes—promoting metabolic diversity and resilience [29]. When keystones are lost (e.g., after antibiotics), colonization resistance weakens and opportunists such as *difficile* can dominate [30].

*F. prausnitzii* is a major butyrate-producing Firmicute [31]; butyrate supports epithelial barrier integrity and host homeostasis [5,32] and *F. prausnitzii* exerts anti-inflammatory effects, including a secreted MAM protein that suppresses NF-κB and protects in colitis models [28,33]. Its presence, thereby, favors low-inflammation, anaerobe-friendly conditions (i.e., conditions that do *not* promote the oxygen/nitrate influx that drives Enterobacteriaceae blooms) [34]. Loss of *F. prausnitzii* is associated with Crohn’s disease dysbiosis [28].

*A. muciniphila* is a mucin-degrading bacterium that resides in the mucus layer [35,36]. It supports mucus-layer homeostasis and can increase mucus thickness/production, strengthening barrier function in vivo [36,37]. By liberating mucin-derived oligosaccharides, it underpins cross-feeding at the mucosal interface and contributes to host–microbe mutualism that favors mucin-adapted commensals and helps deter pathogen overgrowth via improved barrier/colonization resistance [38].

*B. thetaiotaomicron* is a polysaccharide specialist equipped with numerous polysaccharide utilization loci (PULs) that sense and deconstruct diverse dietary and host glycans [39]. By initiating complex glycan digestion, it releases simpler metabolites that other microbes consume, promoting metabolic diversity and community flexibility to diet shifts [40]. When key glycan specialists are depleted, trophic chains are perturbed and colonization resistance weakens—opportunists such as *Clostridioides difficile* can dominate [30]. Such dysbiosis can involve reduced SCFA output, altered redox/oxidative stress and mucus-layer damage, while the community becomes less resilient to perturbations (e.g., antibiotics, diet) [5,41,42].

### 2.4. Ecological Succession and Resilience

The microbiome evolves over time (e.g., infancy to adulthood) through stages of colonization, often dictated by pioneer species. Communities exhibit resilience by reestablishing structure after disruptions (e.g., antibiotic use), largely due to microbial interdependence.

Early Life Microbiome Development (Succession). Newborn guts are initially colonized by facultative anaerobes (often Enterobacteriaceae) and then transition toward obligate anaerobes such as *Bifidobacterium*, *Bacteroides*, and *Clostridia* as oxygen is consumed and diet changes; mode of delivery further shapes these trajectories [43,44].

Antibiotic-Induced Disturbance (Resilience). Broad-spectrum antibiotics sharply reduce diversity and butyrate producers, enabling pathogen blooms; communities can partially recover within weeks to months, though recovery is often incomplete and individualized [42]. In recalcitrant dysbiosis (e.g., recurrent *C. difficile*), fecal microbiota transplantation can restore homeostasis [45].

Dietary Shifts and Microbiome Plasticity. High-fiber diets enrich SCFA-producing genera such as *Roseburia* and *Faecalibacterium*, supporting barrier function and anti-inflammatory tone. Conversely, animal-based/high-fat patterns rapidly (≤48 h) increase bile-tolerant microbes (*Bilophila*, *Bacteroides*, *Alistipes*) and decrease fiber-adapted *Firmicutes*; milk-fat-driven bile acids specifically promote *Bilophila wadsworthia* and colitis in susceptible hosts. The microbiome often reverts toward prior states when fiber is reintroduced, illustrating ecological resilience [46].

Recent evidence indicates that oxidative stress acts as an ecological disturbance driver in the intestinal ecosystem. Nanoplastic exposure increases reactive oxygen species (ROS) in epithelial and microbial cells [47,48] and thereby alters redox potential, oxygen diffusion, and nutrient gradients across the mucosal surface. In ecological terms, this functions like a pulse disturbance that resets community succession: oxygen-tolerant and ROS-resistant taxa (often Gram-negative *Proteobacteria* and *Bacteroidetes*) become early colonizers of the oxidized niche, while strict anaerobic *Firmicutes*—key butyrate producers—decline temporarily. If oxidative pressure persists, the system may stabilize in a new, less diverse state dominated by stress-tolerant opportunists. When the stress subsides, recovery follows a secondary succession pattern in which antioxidant-producing commensals and mucin degraders (*Akkermansia*, *Faecalibacterium*) recolonize and restore anaerobic conditions. Integrating this dynamic model of redox-driven microbial succession couples ecological resilience theory with the mechanistic nano-toxicology framework developed in later sections.

In summary, succession refers to predictable, staged development or turnover of microbial communities (e.g., during early life or after a major perturbation). And resilience reflects the microbiome’s ability to bounce back to a stable, functional state after disturbances like antibiotics, infection, or diet change. The intestinal microbiome is a finely balanced web of microbial life where cooperation, competition, and cross-feeding underpin stability and function. Understanding these relationships is crucial for manipulating the microbiome for health benefits, such as through diet, probiotics, or microbiota transplantation.

## 3. Interaction of MNP and Bacteria

**Figure 2:**
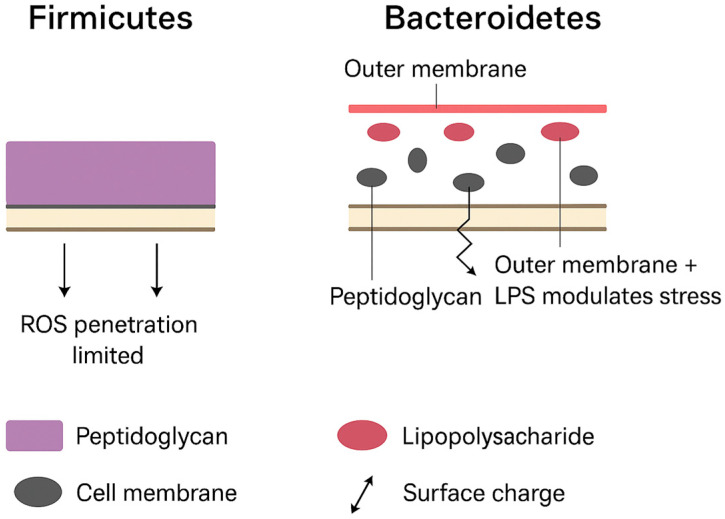
Structural differences between *Firmicutes* and *Bacteroidetes* shape their responses to nanoplastic-induced oxidative stress. *Firmicutes* (**left**) possess a thick peptidoglycan layer without an outer membrane, which restricts penetration of reactive oxygen species (ROS) and confers relative resistance to oxidative injury. In contrast, *Bacteroidetes* (**right**) have a thinner peptidoglycan wall surrounded by an outer membrane enriched in lipopolysaccharides (LPS). This outer membrane contributes to surface charge interactions and modulates ROS stress responses but also provides points of vulnerability to nanoparticle adsorption and penetration. These structural distinctions help explain why nanoplastic exposure often results in disproportionate depletion of *Firmicutes* and relative enrichment of *Bacteroidetes*.

The encounter of MNPs with bacteria entails their binding to the cell wall, the damage caused by gaining entry(including electrostatic), the impact of size redox effects, biofilm effects, and the resulting oxidative stress (Figure 2). These three areas of interaction are discussed in the next sections.

### 3.1. Binding and Physical Membrane Disruption

Binding of MNPs can physically damage the cell membrane and result in membrane permeability. MNPs can directly interact with bacterial cell envelopes, often through physical adsorption to the cell surface. This attachment can impose mechanical stress on membranes: micrometer-sized plastic beads (1–10 μm) binding to lipid bilayers cause significant membrane stretching even without any chemical or oxidative reactions [49]. Irregularly shaped microplastics (MPs) with sharp edges and high curvature can physically damage cell membranes upon contact [50]. Such mechanical perturbation increases membrane tension and can create nano-scale pores, elevating permeability without immediately lysing the cell [51]. For instance, 60 nm PS were shown to induce membrane permeabilization while maintaining overall bilayer integrity [52]. These findings align with model membrane studies in which plastic particles increase membrane tension and reduce cell membrane stability/lifetime [49].

### 3.2. Electrostatic Interactions and Surface Charge Effects

Electrostatic interactions represent another key mechanism of MNP-induced bacterial stress. Bacterial cell walls are generally negatively charged—due to peptidoglycan in Gram-positives and lipopolysaccharide (LPS) in Gram-negatives—so positively charged nanoplastics bind more readily. For example, amino-functionalized (cationic) 30 nm polystyrene nanoparticles attached strongly to bacterial cells and elevated intracellular oxidative stress. In contrast, neutral 30 nm PS caused both oxidative damage and physical membrane disruption [51]. Negatively charged nanoparticles (e.g., carboxylated) can still adsorb through hydrophobic and van der Waals forces, altering bacterial surface charge [51]. Zeta potential studies further confirm that 100 nm PS beads, which are slightly negative, coat both *S. aureus* (Gram-positive) and *Klebsiella pneumoniae* (Gram-negative). This coating reduces the net negative surface charge but does not directly kill the cells.

Significant zeta potential shifts (signifying particle binding) only occurred at concentrations of PS ≥ 20 µg/mL for *S. aureus*, whereas *Klebsiella* (Gram-negative) showed surface charge changes even at 2–20 µg/mL [51]. The authors attributed this to the thicker peptidoglycan layer of Gram-positive cells, which may initially limit nanoparticle access. Nonetheless, at higher doses (100 µg/mL), *S. aureus* did bind many PS particles, resulting in an even greater increase in net negative surface charge (due to blocking of positively charged sites). Atomic force microscopy (AFM) images showed PS beads decorating the *S. aureus* surface, but again no obvious cell wall breakage. This suggests that Gram-positive cell walls can accommodate a fair amount of adsorbed plastic without immediate rupture. However, subtle damage can occur with positively charged nanoparticles, creating ion-selective pores in the membrane that could dissipate the proton motive force of Gram-positive species. Such pore formation in *Firmicutes* would increase cell wall permeability (to ions and small molecules) and could be detected by uptake of viability dyes (e.g., propidium iodide) even if the cell is not lysed.

A recent in vivo study found that *Lachnospiraceae*, a family in *Firmicutes*, readily took up 100 nm PS particles in the gut [53]. Confocal imaging confirmed nanoplastic accumulation inside fecal bacteria shortly after exposure. Although specific cell wall damage in those bacteria was not microscopically detailed, the internalization implied that the plastic either traversed or seriously compromised the cell envelope. Positively charged nanoplastics are known to bind avidly to negatively charged Gram-positive bacterial surfaces (e.g., *B. subtilis*) and often cause significant membrane damage [54,55]. Neutral or negatively charged particles typically show far less membrane perturbation. For example, neutral/negative 100 nm PS particles adhered to the cell membranes of *S. aureus*, but they did not compromise the membrane integrity. They did, however, inhibit bacterial growth at 50 mg/mL [51]. Kim et al. reported that 100 mg/L 60 nm negatively charged PS attached to the cell surfaces of Gram-positive *Bacillus* and *Lactobacillus*. They entered cells and caused growth inhibition [56].

Zhao et al. showed that another Gram-positive bacterium (*Lactobacillus*) took up polypropylene (PP), polyethylene (PE), polyvinyl chloride (PVC) [57]. Neutral or negatively charged nanoplastic also generated ROS and caused oxidative damage in *Bacillus* [56,58]. They also altered membrane properties and enzyme activities [59] and even modulate bacterial growth and gene expression. This reinforces that even without a positive charge, nanoplastics can impact Gram-positive microbes in the gut environment. Some *Firmicutes*, like *Ruminococcaceae*, interacted with NPs to a lesser extent, and some (e.g., certain butyrate producers) might be inhibited if their cell envelope is disturbed.

Overall, in vitro experiments illustrate that Gram-positive gut bacteria can adsorb or even internalize micro/nanoplastics while surviving, often by mounting stress responses (antioxidant production, biofilm formation). Yet prolonged exposure can skew the Firmicute community, likely by impairing more sensitive members’ membrane functions or by favoring species that capitalize on plastic surfaces (the so-called “plastisphere” colonizers).

### 3.3. Particle Size and Penetration

Particle size plays a critical role in the depth of interaction. Smaller nanoplastics (<100 nm) have higher surface-area-to-volume ratios and can interact more intimately with cell membranes. They exhibit enhanced Brownian motion and often penetrate closer to or even into cell envelopes [58]. Indeed, 30 nm polystyrene (PS) nanoparticles were suggested to penetrate bacterial membranes and possibly enter cells, disrupting membrane proteins [51]. Such nano-scale plastics (NPs) can lodge within the cell wall or periplasm of Gram-negative bacteria, compromising envelope integrity. Wang et al. also noted that generally “interactions between bacterial cells and nanoplastics result in membrane rupture” along with downstream effects like loss of hydrolase enzyme activity and changes in surface charge [58]. In other words, prolonged or high-concentration NP exposure can physically breach the cell envelope. By contrast, larger microplastics (several microns) usually remain external but can still cause abrasions and micro-tears in the cell wall upon contact [50], or induce the cell to stretch around the particle [49]. Notably, shape and surface topology influence this: irregular or fiber-shaped plastics (e.g., fragmented PET fibers) can pierce or insert into bacterial cell walls more readily than smooth spheres [50].

### 3.4. Oxidative Stress (Host and Bacterial Endpoints)

In the studies reviewed, oxidative stress endpoints were measured using standard host antioxidant defense systems, most commonly superoxide dismutase (SOD), catalase (CAT), and glutathione peroxidase (GSH-Px), together with malondialdehyde (MDA) as a marker of lipid peroxidation. These assays were performed in the intestinal tissue of mouse and zebrafish models following oral gavage of polystyrene nanoplastics (50–100 nm) at sub-chronic doses (14–60 days). Results consistently showed that nanoplastic exposure reduced SOD, CAT, and GSH-Px activity while elevating MDA levels [60,61]. These biochemical changes were paralleled by microbial compositional shifts, viz. depletion of SCFA-producing *Firmicutes* and enrichment of Gram-negative taxa. Such changes suggest that antioxidant system impairment and microbiome disruption may act in concert to exacerbate epithelial barrier dysfunction. Dose–response relationships were also observed, with higher NP concentrations producing more pronounced suppression of antioxidant enzymes [62].

*Bacteroides* spp. possess several mechanisms that allow survival in moderately oxidizing conditions. They produce enzymes like SOD, catalase, and peroxidases, which help detoxify ROS. Some strains can upregulate antioxidant defense genes under oxidative stress, maintaining metabolic function. They can shift metabolic strategies (e.g., using alternative electron acceptors) that allow persistence when redox conditions fluctuate. Thus, in a gut under oxidative stress, *Bacteroides* may face less competition from ROS-sensitive taxa (e.g., *Clostridia*/*Firmicutes*), outgrowing other microbes, and increasing their relative abundance. [63].

Besides mechanical interactions, chemical and redox processes contribute to cell wall damage. Broader research outlines oxidative stress pathways as a common response mechanism to nanoparticle exposure across microbes, highlighting excess reactive oxygen species (ROS) generation as a key toxicity pathway [64]. Many studies report that MNPs elicit elevated ROS in bacteria, which in turn damages membrane lipids and proteins [58]. Oxidative stress can peroxidize the lipid components of the cell membrane, increasing permeability. Studies on MNP polystyrene particles report that they were internalized by *E. coli* and *Bacillus* sp., triggering ROS production in both bacterial types [56,65].

Antioxidant defenses in bacteria (e.g., superoxide dismutase, catalase) are often upregulated upon microplastic exposure as a response to ROS-induced damage. In one experiment, exposing a tilapia gut *Bacillus* (*Firmicutes*) to PS MP provoked a spike in intracellular ROS, forcing the bacteria to increase SOD and catalase levels [66]. If ROS overwhelm defenses, they can compromise the cell wall: lipid peroxidation makes the membrane leaky and can trigger autolytic pathways. Thus, ROS-mediated oxidative damage is a common mechanism by which plastics harm bacterial membranes, often acting in tandem with direct contact effects.

### 3.5. Biofilm Interactions

Micro/nanoplastics can impact bacterial biofilm formation and structure, indirectly affecting cell wall integrity. When MNPs are present, bacteria may alter exopolysaccharide (EPS) production, either increasing biofilm matrix to sequester and immobilize the foreign particles or experiencing disrupted biofilm cohesion. Some studies show enhanced biofilm formation; for instance, *Pseudomonas aeruginosa* exposed to sub-lethal PS nanoplastics produced more EPS and formed thicker biofilms, a response linked to nanoparticle-induced ROS signaling [67]. Similarly, gut-derived *Bacillus tropicus* responded to PS microplastics by overproducing EPS and actively colonizing the plastic surfaces. Scanning electron microscopy images confirmed *Bacillus* cells densely adhered to irregular PS fragments, encased in biofilm, which likely protects the cells’ walls from direct contact damage [66]. On the other hand, plastics can also disrupt existing biofilms by adsorbing bacteria and disturbing their normal attachment to gut mucosa or other surfaces. A 2022 in vitro digestion study found that PET microplastics introduced into human gut microbial communities became coated with bacteria, suggesting microbes were pulling off from their usual niches to cling to the plastic particles [68]. The authors hypothesize this could alter biofilm architecture and microbial spatial organization in the gut. In summary, MNPs can trigger bacteria to modify their cell wall environment—either by excess matrix production as a defense or by inadvertently causing biofilm destabilization—both of which reflect an impact on the integrity and function of bacterial cell walls.

Experimental studies in animals (e.g., mice, fish, rabbits) exposed to microplastics consistently show shifts in the microbiome. Papp et al. reported that at higher PVC levels, an alteration in the gut microbiota composition, with increased *Bacteroidetes* and decreased *Firmicutes* [69]. Galecka and coworkers reported a decrease in *Firmicutes* (often strict anaerobes sensitive to oxidative conditions), and a relative increase in *Bacteroidetes*, including *Bacteroides* spp. [70]. *Bacteroides* species—especially *B. thetaiotaomicron*—can adapt metabolically to reduce ROS internally. Thus, under oxidative pressure, *Bacteroidetes* are more likely to dominate due to enhanced survival and flexibility [71]. This pattern may support the idea that oxidative pressure favors *Bacteroidetes* by weakening redox-sensitive competitors and selecting for metabolically flexible, ROS-tolerant bacteria.

## 4. Materials and Methods

A systematic, comparative review of the literature is difficult because there are no studies that include a side-by-side comparison of different plastics in the same species, using the same analysis of dysbiosis, bacteria, and gut health.

A total of eleven reviews were reviewed and verified by the authors for accuracy and integrity (see below, Section 4.3. Flow of Evidence Selection and Table 1). With the aid of AI (ChatGPT 5), we screened candidate primary studies for integrity concerns using a forensic checklist adapted from Aquarius et al. [72] and Abalkina et al. [73], which includes image duplication, statistical anomalies, textual boilerplate, venue risk, and evidence of salami slicing. This approach follows established science-sleuth methodology [74,75].

### 4.1. Eligibility Criteria

Studies were considered eligible if they (1) were published in peer-reviewed journals between 2000 and mid-2025, (2) were written in English, and (3) investigated the effects of microplastics (≤5 mm) or nanoplastics (≤100 nm) on the gut microbiome and/or intestinal health. Eligible exposures included oral or dietary administration of pristine or environmentally aged particles of common polymers (PS, PE, PP, PVC, polyethylene terephthalate (PET), or mixtures thereof). Both primary studies (in vivo animal models, in vitro bacterial or epithelial systems, ex vivo digestion simulations) and review articles were included if they reported outcomes related to gut microbiota composition (e.g., 16S rRNA sequencing, metagenomics, diversity metrics, *Firmicutes*/*Bacteroidetes* ratio) and/or gut epithelial effects (e.g., tight junction integrity, reactive oxygen species, intestinal permeability, mucus secretion, apoptosis, or inflammation). Studies were excluded if they (1) lacked microbiome or intestinal endpoints, focusing exclusively on other organ systems (e.g., liver, brain, placenta); (2) reported only environmental distribution, degradation, or detection of plastics without biological exposure models; (3) were preprints, conference abstracts, or other gray literature not peer-reviewed; or (4) consisted solely of material science or particle characterization without biological assays. This was accomplished using the Tables S2–S4 in Supplemental Information. The summaries and population were assisted by AI and checked for accuracy by the authors.

### 4.2. Information Sources and Search Strategy

A comprehensive literature search was conducted to identify peer-reviewed studies and reviews published between January 2000 and mid-2025. Electronic databases included PubMed, Scopus, Web of Science, ScienceDirect, SpringerLink, Wiley Online Library, and Taylor & Francis Online. Additional searches were performed in Google Scholar to capture items not indexed in the major databases. The search strategy combined terms for plastics with microbiome- and gut health-related concepts. Core terms included “microplastic,” “nanoplastic,” “plastic particle,” “polystyrene,” “polyethylene,” “polypropylene,” “polyvinyl chloride,” “polyethylene terephthalate” in combination with “gut,” “intestine,” “microbiome,” “microbiota,” “dysbiosis,” “*Firmicutes*,” “*Bacteroidetes*,” “intestinal barrier,” “tight junction,” “reactive oxygen species,” and “inflammation.” Search strings were adapted for the syntax of each database. No restrictions were placed on host species at the search stage. All retrieved records were imported into a reference manager, and duplicates were removed. Screening proceeded with the aid of AI in two stages: first by title and abstract, and then by full-text review against the eligibility criteria. Reference lists of relevant reviews were also hand-searched to identify additional studies.

### 4.3. Flow of Evidence Selection

The 7300 records across PubMed, Scopus, Web of Science, and other databases (see Table S1) were identified through AI-assisted database searching and additional hand-searching of relevant reviews. After removal of duplicates and screening of titles and abstracts, approximately 250 full-text articles were assessed for eligibility. Of these, 166 were excluded for not meeting the inclusion criteria (e.g., no gut microbiome or intestinal outcomes, non-biological studies, preprints), resulting in the exclusion of ~7050 records that did not meet eligibility criteria. Ultimately, 56 studies were included in the scoping review: 11 including review articles (2020 to mid-2025) and 47 primary studies (2020 to mid-2025), with the addition of key earlier studies [47,76].

### 4.4. Data Charting Process

Data from each included study were charted independently by one reviewer and verified by a second to ensure accuracy. A standardized charting form was developed and piloted on a subset of studies before full extraction. The form captured bibliographic details (first author, year, journal, DOI), study design (in vivo, in vitro, ex vivo, review), host species or model system, particle type (polymer, size, surface modification), exposure route and duration, dose, and primary outcomes. Outcomes included gut microbiome composition (taxonomic shifts at phylum, family, or genus levels, *Firmicutes*/*Bacteroidetes* ratio, diversity indices), epithelial or immune endpoints (tight junction proteins, reactive oxygen species, apoptosis, mucus secretion, cytokine expression), and systemic outcomes (metabolic changes, hepatic lipid metabolism). For reviews, charting captured the scope (human, animal, in vitro evidence), reported outcomes (barrier integrity, oxidative stress, dysbiosis), and main conclusions. Any discrepancies in data charting were resolved through discussion and consensus.

### 4.5. Data Items

From each included study, we extracted the following variables:

- Bibliographic details: first author, year of publication, journal, DOI.
- Study design: in vivo (animal model), in vitro (bacterial or epithelial system), ex vivo (digestion or fermentation model), or review.
- Host/model: species (mouse, zebrafish, rabbit, chicken, turtle, bacterial culture, cell line).
- Particle characteristics: polymer type (polystyrene, polyethylene, polypropylene, PVC, PET), size classification (microplastic ≤ 5 mm, nanoplastic ≤ 100 nm), surface properties or modifications, and whether pristine or environmentally aged.
- Exposure details: route (oral gavage, diet, drinking water), duration of exposure, and dose/concentration.
- Microbiome outcomes: sequencing method, phylum/family/genus-level taxonomic changes, *Firmicutes*/*Bacteroidetes* ratio, alpha/beta diversity indices, presence of key functional taxa (e.g., SCFA producers, mucin degraders, endotoxin producers).
- Barrier and immune outcomes: expression of tight junction proteins (occludin, claudins, ZO-1), mucus secretion, epithelial apoptosis, reactive oxygen species (ROS), pro- and anti-inflammatory cytokine levels, immune gene expression.
- Systemic outcomes: host metabolic alterations (e.g., hepatic lipid metabolism, glucose regulation), extra-intestinal immune responses.
- Review-specific items: stated scope (humans, animals, in vitro), thematic focus (barrier, oxidative stress, dysbiosis), and main conclusions.

### 4.6. Gram-Positive/Gram-Negative Weighting Strategy

To systematically evaluate how nanoplastic exposure influenced the relative balance of Gram-positive and Gram-negative bacteria, we developed a standardized weighting strategy. For each study, reported taxa shifts at the phylum or genus level were classified by Gram status, and directional changes were tallied. Gram-positive taxa (e.g., *Firmicutes*, *Lactobacillus*, *Bifidobacterium*, *Actinobacteria*) and Gram-negative taxa (e.g., *Bacteroidetes*, *Proteobacteria*, *Escherichia-Shigella*, *Akkermansia*) were included when authors reported significant increases or decreases.

Each significant increase in a Gram-positive taxon contributed +1 to the “Gram+ ↑ (w)” column, while each significant decrease contributed +1 to the “Gram+ ↓ (w)” column. Analogously, increases in Gram-negative taxa contributed +1 to the “Gram− ↑ (w)” column, and decreases contributed +1 to the “Gram− ↓ (w)” column. In cases where multiple taxa within the same phylum were reported, each genus or family was scored individually. Where results within a phylum were inconsistent, the score was weighted toward the dominant or statistically significant direction.

The weighted net outcome was then derived by integrating these tallies:

- When losses of Gram-positive commensals coincided with gains in Gram-negative taxa (Gram+ ↓ + Gram− ↑), the net was recorded as “↓ Gram+/Gram− (dominant Gram− expansion).”
- When increases in Gram-positive taxa coincided with losses in Gram-negative taxa (Gram+ ↑ + Gram− ↓), the net was recorded as “↑ Gram+/Gram− (commensal enrichment, pathogen reduction).”
- When tallies were balanced or shifts were nonsignificant, the net was recorded as “No net effect.”

This weighting system ensured that studies reporting multiple genus-level changes did not collapse into a single phylum-level shift and allowed consistent cross-study comparison of the directional trends in Gram-positive versus Gram-negative responses to nanoplastic exposure (Table S5).

### 4.7. Critical Appraisal of Sources of Evidence

Consistent with the objectives of a scoping review, we did not exclude studies on the basis of methodological quality. However, we noted several recurring issues relevant to the interpretation of findings:

- Dose levels: Many in vivo studies used high doses of microplastics (tens to hundreds of mg/day per mouse) that exceed environmentally relevant exposures, limiting translational inference.
- Particle characterization: Incomplete reporting of polymer identity, particle size distribution, or surface properties was common.
- Microbiome analysis: Several studies reported phylum-level changes (e.g., *Firmicutes*/*Bacteroidetes* ratio) without deeper taxonomic resolution or appropriate compositional analysis.
- Reproducibility and overlap: Some clusters of publications (e.g., [47,77,78]) showed methodological redundancy, raising concerns about salami-slicing, while other groups (e.g., [53,79]) used more rigorous, environmentally relevant models.
- Venue and integrity risk: Journals such as *Bioengineered* have been flagged for paper-mill infiltration, but none of the studies included here came from that venue. All included studies were screened against the “Aquarius checklist” to minimize the risk of incorporating unreliable evidence [72].

We therefore present all eligible studies to map the breadth of available evidence, while highlighting methodological limitations and integrity concerns in the synthesis.

### 4.8. Selection of Sources of Evidence

As described in Section 4.3. Flow of Evidence Selection, eleven reviews summarized evidence on micro- and nanoplastic effects on gut microbiota and intestinal health, and 47 primary studies, most published between 2020 and mid-2025, were reviewed. In addition, some key earlier work (e.g., [47,76]) was reviewed to provide historical context on microbiota disruption and barrier function.

The flow of information through the selection process is presented in Figure 2. PRISMA-ScR flow diagram of study selection (*n* = 56 included studies).

## 5. Results

### 5.1. Characteristics of Sources of Evidence

**Figure 3:**
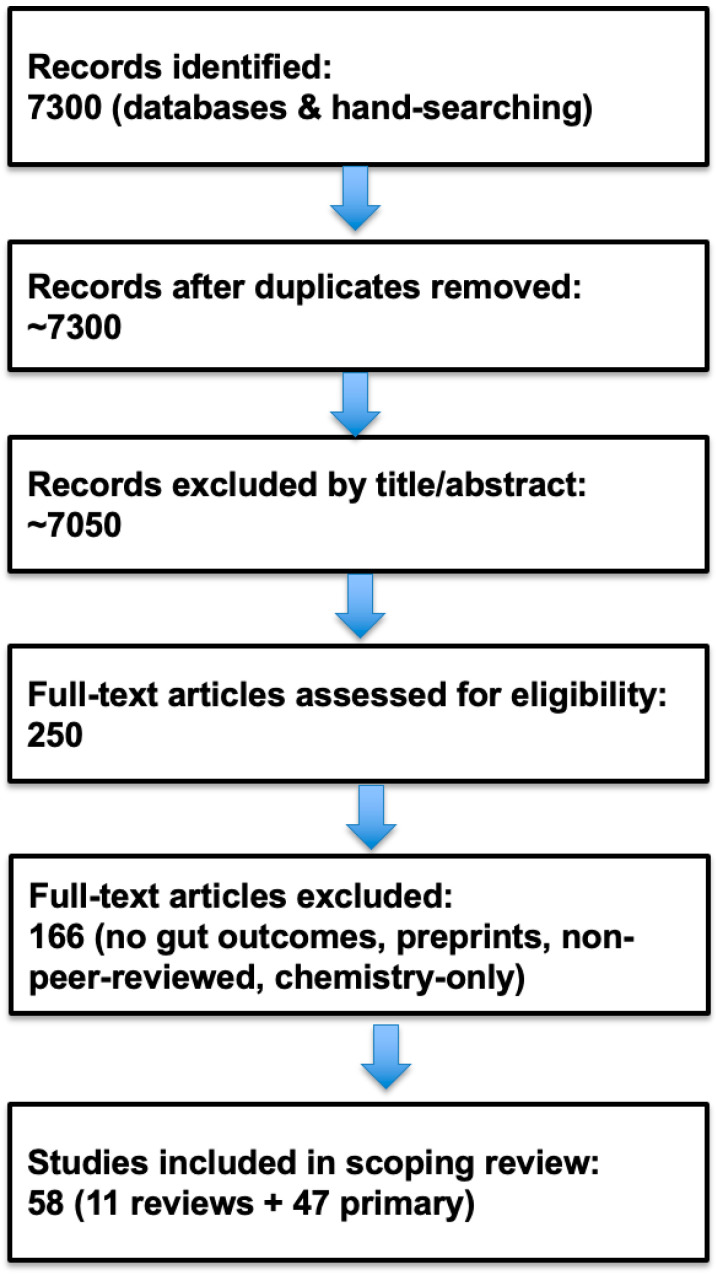
PRISMA-ScR flow diagram of study selection for the scoping review.

The 56 included studies comprised a mix of review articles (*n* = 11) and primary research studies (*n* = 47) (Figure 3). Review articles, published between 2020 and mid-2025 (mostly 2024), varied in scope from human-focused reviews of exposure and health outcomes to mechanistic discussions of barrier disruption, oxidative stress, and microbiome dysbiosis.

Certain characteristics/parameters among the primary studies are detailed in Table S2 and summarized here:

- Host species and models included mice, zebrafish, chickens, rabbits, turtles, bacterial cultures, gut epithelial cell lines, and in vitro/ex vivo digestion models.
- Particle types most frequently investigated were polystyrene (PS), polyethylene (PE), polypropylene (PP), polyvinyl chloride (PVC), and polyethylene terephthalate (PET).
- Particle sizes ranged from microplastics (10–5000 µm) to nanoplastics (≤100 nm), with some studies comparing different size fractions or surface modifications (carboxyl, amine).
- Exposure routes and durations included oral gavage, dietary supplementation, and drinking water administration, ranging from short-term (7 days) to chronic exposures (up to 12 weeks).
- Doses varied widely, from low environmentally relevant levels (100 µg/day) to high experimental doses (tens to hundreds of mg/days per mouse).
- Outcomes measured encompassed gut microbiome composition (phylum- to genus-level taxonomic shifts, diversity metrics, *Firmicutes*/*Bacteroidetes* ratio, presence of key functional taxa such as mucin degraders, SCFA producers, or endotoxin-associated groups), intestinal barrier integrity (tight junction proteins, mucin production, epithelial apoptosis, permeability), immune signaling and inflammatory markers (cytokine expression, ROS, apoptosis, immune gene regulation), and systemic outcomes (hepatic lipid metabolism, glucose homeostasis).

Additional details for each included study are provided in Table 1 (reviews), Tables S2–S4 (primary studies) in the SI.

### 5.2. Results of Individual Sources of Evidence

To provide a clearer scientific structure, the evidence was organized into thematic groups reflecting the principal mechanisms by which micro- and nanoplastics (MNPs) affect the intestinal system:

(a) Epithelial barrier injury;
(b) Oxidative stress and immune activation;
(c) Microbiome compositional and functional changes;
(d) Direct bacteria–particle interactions.

Each subsection integrates findings from both review articles and primary studies.

#### 5.2.1. Epithelial Barrier Injury

Across multiple in vivo mammalian and fish models, MNP exposure consistently impaired intestinal barrier integrity. In mice, Lu 2018 [47], Jin 2019 [77], and Li 2020 [78] reported reduced tight-junction proteins (occludin, ZO-1, claudins), villus shortening, and increased permeability. Similar findings were observed in zebrafish after PS-NP exposure (Rehman 2025 [61]). The severity of barrier loss scaled with particle size and dose. In contrast, low-dose, environmentally relevant PET fragments produced only subtle immune modulation without overt barrier injury (Harusato 2023 [79]), indicating a threshold below which structural damage is limited. Reviews by Covello 2024 [7], Popa 2025 [14], and Wang 2024 [16] all emphasized the same mechanism, linking barrier dysfunction to subsequent dysbiosis.

#### 5.2.2. Oxidative Stress and Immune Activation

Seventeen studies quantified antioxidant and inflammatory endpoints. PS-NP exposure (50–100 nm, 14–60 days) consistently lowered superoxide dismutase (SOD), catalase (CAT), and glutathione peroxidase (GSH-Px) while elevating malondialdehyde (MDA) (Lu 2018 [47]; Xie 2022 [80]; Rehman 2025 [61]). These biochemical shifts paralleled activation of proinflammatory cascades (NF-κB, TLR4, IL-6, TNF-α) and barrier disruption. Mechanistic reviews (Kurhaluk 2025 [12], Li 2024 [81]) highlighted oxidative stress as a unifying pathway operating through Nrf2/Keap1, MAPK, and JAK/STAT signaling. Collectively, oxidative imbalance emerges as the central mediator linking nanoplastic exposure to intestinal inflammation.

#### 5.2.3. Microbiome Composition and Function

Thirty-six studies (twenty-seven in vivo, nine in vitro/ex vivo) reported microbial outcomes.

- Phylum level: A downward *Firmicutes*/*Bacteroidetes* (F/B) ratio was documented in most nanoplastic exposures (Lu 2018 [47]; Jin 2019 [77]; Li 2020 [78]; Xie 2022 [80]; Su 2024 [63]; Hsu 2025 [53]; Rehman 2025 [61]).
- Functional taxa: Depletion of SCFA-producing *Firmicutes* (e.g., *Faecalibacterium*, *Roseburia*) and mucin-degrading taxa (*Akkermansia muciniphila*) coincided with enrichment of Gram-negative groups such as *Prevotellaceae* and *Proteobacteria*.
- Dose dependence: High-dose PS or PE produced pronounced dysbiosis, whereas low-dose PET caused only minor shifts (Harusato 2023 [79]). Recent reviews (Bora 2024 [6]; Eichinger 2024 [9]) independently reached the same conclusion—that functional taxa provide stronger mechanistic insight than the phylum-level F/B ratio.

#### 5.2.4. Direct Bacteria–Particle Interactions

Microscopy-based studies demonstrated that MNPs physically associate with bacterial cell envelopes. Dai 2022 [54], Kim 2022 [56], Zajac 2023 [51], and Perez 2025 [55] showed polystyrene nanoparticles adhering to or entering bacterial membranes, altering zeta potential and inducing reactive oxygen species. Gram-positive *Firmicutes* (e.g., *Bacillus*, *Lactobacillus*) displayed sub-lethal envelope stress and occasional internalization, whereas Gram-negative models (*E. coli*, *Klebsiella pneumoniae*) exhibited outer-membrane adsorption and transient permeability. These laboratory findings support in vivo observations of *Firmicutes* decline and Gram-negative enrichment (Hsu 2025 [53]; Rehman 2025 [61]).

### 5.3. Synthesis of Results

The collective evidence across 56 studies (11 reviews and 47 primary investigations) reveals convergent mechanisms through which micro- and nanoplastics (MNPs) impair intestinal homeostasis. When organized thematically, three interconnected pathways emerge: oxidative stress, barrier dysfunction, and microbial imbalance.

#### 5.3.1. Oxidative Stress as the Initiating Event

Most nanoplastic exposures, particularly with 50–100 nm polystyrene, triggered significant oxidative stress in intestinal tissue and microbial cells. Key antioxidant enzymes (SOD, CAT, GSH-Px) were consistently suppressed, whereas lipid-peroxidation marker MDA rose sharply (Lu 2018 [47]; Xie 2022 [80]; Rehman 2025 [61]). Mechanistic reviews (Kurhaluk 2025 [12]; Li 2024 [81]) link these findings to activation of Nrf2/Keap1 and NF-κB signaling, confirming oxidative imbalance as the upstream driver of cellular and microbial injury.

#### 5.3.2. Barrier Disruption and Inflammation

Oxidative damage corresponded closely with epithelial pathology. Mouse and zebrafish models showed decreased expression of tight-junction proteins (occludin, claudins, ZO-1), goblet-cell loss, and elevated cytokines (Lu 2018 [47]; Jin 2019 [77]; Li 2020 [78]; Su 2024 [63]; Rehman 2025 [61]). At environmentally realistic doses, PET particles produced only minor immune modulation (Harusato 2023 [79]), defining a probable threshold below which inflammation remains subclinical. Together, these data confirm that epithelial compromise is a secondary—but pivotal—consequence of redox imbalance.

#### 5.3.3. Microbiome Reconfiguration and Functional Loss

Most in vivo studies reported a reproducible decrease in the *Firmicutes*/*Bacteroidetes* (F/B) ratio and a parallel decline in the Gram+/Gram− balance. Depletion of SCFA-producing *Firmicutes* (*Faecalibacterium*, *Roseburia*) and mucin degraders (*Akkermansia muciniphila*) was accompanied by enrichment of Gram-Negative taxa (*Prevotellaceae*, *Proteobacteria*) (Hsu 2025 [53]; Rehman 2025 [61]). Functional loss of butyrate and mucus-supporting pathways links directly to the barrier defects described above. Reviews (Bora 2024 [6]; Eichinger 2024 [9]) emphasize that these functional shifts, rather than phylum-level ratios alone, provide the most reliable indicators of dysbiosis.

#### 5.3.4. Integrative Model

Across taxa and host systems, MNP exposure yields a reproducible triad:

1. Oxidative stress—initiated by nanoparticle–cell interactions;
2. Barrier disruption—loss of tight-junction integrity and mucosal protection;
3. Microbiome shift—preferential loss of Gram-positive commensals and overgrowth of Gram-negative, stress-tolerant taxa.

Only low-dose PET exposures (Harusato 2023 [79]) fell outside this sequence, suggesting a threshold effect. These linked outcomes define a unified mechanistic framework: oxidative stress destabilizes the epithelium, the weakened barrier alters oxygen and nutrient gradients, and this new microenvironment favors Gram-Negative expansion, reflected as a decrease in the F/B and Gram+/Gram− ratios. This model integrates molecular, microbial, and ecological perspectives into a coherent understanding of nanoplastic-induced dysbiosis.

### 5.4. Summary of Evidence

The evidence base coherently supports a mechanistic cascade in which nanoplastic exposure initiates oxidative stress, compromises epithelial integrity, and reconfigures the intestinal microbiome. Across mouse and zebrafish studies (Lu 2018 [47], Jin 2019 [77], Li 2020 [78], Xie 2022 [80]; Hsu 2025 [53]; Rehman 2025 [61]), antioxidant depletion (↓ SOD, CAT, GSH-Px; ↑ MDA) preceded loss of tight-junction proteins and subsequent declines in *Firmicutes*/*Bacteroidetes* and Gram+/Gram– ratios. Thematically grouped evidence from Section 5.2 and Section 5.3, therefore, indicates a reproducible triad of oxidative stress → barrier dysfunction → microbiome imbalance. Functional taxa changes—loss of SCFA producers (*Faecalibacterium*, *Roseburia*) and mucin degraders (*Akkermansia*)—provide stronger mechanistic resolution than phylum-level ratios alone. Only environmentally realistic, low-dose PET exposures (Harusato 2023 [79]) departed from this pattern, underscoring a probable threshold for dysbiosis. Overall, the thematic synthesis highlights that nanoplastics compromise intestinal homeostasis through integrated oxidative, structural, and ecological mechanisms.

## 6. Limitations of the Review Process

Several limitations should be noted in the conduct of this scoping review. First, our search was limited to English-language, peer-reviewed articles, which may have excluded relevant studies in other languages or in the gray literature. Second, although multiple databases and hand searches were used, it is possible that some eligible studies were not retrieved, particularly those indexed under non-standard terminology for plastics or microbiome outcomes. Third, the evidence base itself is highly heterogeneous. Studies varied widely in polymer type, particle size, surface chemistry, exposure duration, host species, and microbiome sequencing methods. These differences preclude direct comparisons and limit the ability to infer dose–response relationships. In addition, many in vivo studies used very high doses of particles relative to environmental exposures, constraining extrapolation to human health contexts. Fourth, this review did not apply a formal risk-of-bias tool, consistent with scoping review methodology. Instead, we screened sources with an “Aquarius checklist” to flag potential integrity issues [72]. While no included studies were retracted or found to contain overt duplications, some clusters [47,77,78] showed methodological overlap, raising concern for salami-slicing. Finally, and most importantly, the *Firmicutes*/*Bacteroidetes* (F/B) ratio—though frequently reported—is a crude indicator of gut microbiome status. Phylum-level changes collapse the complexity of thousands of taxa into a single measure that is sensitive to sequencing pipeline choices, region of the 16S rRNA amplified, and host diet or physiology. An increased or decreased F/B ratio can be interpreted differently depending on context, and it fails to capture the loss of functionally important groups such as SCFA producers or mucin degraders. The reliance of many studies on the F/B ratio as a primary readout may therefore overstate or obscure the true nature of dysbiosis. However, the finding that the weighted Gram+/Gram- ratio also declined supported the decreased F/B ratio (Table S5).

## 7. Conclusions and Implications

This scoping review synthesized evidence from 56 studies (11 reviews and 47 primary investigations) to clarify how micro- and nanoplastics (MNPs) disrupt intestinal homeostasis. When organized thematically, the data reveal a reproducible mechanistic cascade:

1. Oxidative-stress initiation—Nanoplastic exposure, particularly to 50–100 nm polystyrene, consistently induces redox imbalance through excess reactive oxygen species and suppression of antioxidant defenses [47,61,80].
2. Barrier dysfunction—This oxidative injury weakens tight-junction integrity, depletes mucus-producing goblet cells, and elevates inflammatory mediators [63,77,78].
3. Microbiome reconfiguration—The disturbed intestinal milieu favors Gram-negative expansion and loss of Gram-positive commensals, reflected as decreased *Firmicutes*/*Bacteroidetes* and Gram+/Gram− ratios [53,61].

Collectively, these patterns describe a unified oxidative–barrier–microbiome axis through which nanoplastics compromise gut health. Only low-dose, environmentally realistic PET exposures failed to trigger overt dysbiosis, implying a threshold below which homeostatic compensation can occur [79].

Functionally, declines in short-chain-fatty-acid producers (*Faecalibacterium*, *Roseburia*) and mucin degraders (*Akkermansia muciniphila*) weaken epithelial repair and immune regulation, linking compositional shifts directly to physiological outcomes. Thus, decreases in F/B and Gram+/Gram– ratios are not merely taxonomic artifacts but integrated markers of barrier injury and impaired metabolic resilience.

### 7.1. Implications for Future Research

Future investigations should move beyond descriptive dysbiosis toward mechanistic mapping of the nanoplastic–microbiome–host interaction network. To achieve this, several complementary strategies are recommended.

First, experiments must adopt environmentally relevant exposure conditions, including realistic particle concentrations (µg kg^−1^ day^−1^), aged or surface-modified nanoplastics, and chronic low-dose regimens that mirror human intake scenarios [79]. This will help bridge the four-to-five-order-of-magnitude gap between animal and human exposures noted in this review.

Second, studies should employ integrated multi-omics approaches—metagenomics, metatranscriptomics, metabolomics, and host transcriptomics—to link microbial community shifts with epithelial and immune signaling pathways [61,82]. Combining these layers within the same experimental system can reveal causal links between redox imbalance, barrier injury, and metabolic reprogramming.

Third, time-series modeling should be used to capture the temporal succession of microbial and host responses to sustained nanoplastic stress. Such longitudinal designs can distinguish reversible adaptations from irreversible dysbiosis and clarify the kinetics of oxidative stress and barrier repair.

Fourth, cross-species validation—across mammalian, avian, and aquatic models—will identify conserved mechanisms versus host-specific responses [47,53]. Integrating these comparative data into ecological and toxicological frameworks can refine hazard extrapolation to humans.

Finally, advanced in vitro and organoid systems [83] should be combined with in vivo findings to dissect epithelial–microbial crosstalk under controlled redox environments. Parallel use of integrity screening and transparent data-quality controls will reduce redundancy and enhance reproducibility.

Collectively, these approaches will transform current descriptive evidence into a predictive, systems-level understanding of how nanoplastics perturb intestinal ecosystems. Such integration of multi-omics, temporal, and cross-species analyses will be crucial for constructing mechanistic and quantitative risk-assessment frameworks that link oxidative stress, barrier failure, and microbial imbalance to long-term human health outcomes.

### 7.2. Broader Context

Understanding nanoplastic-driven dysbiosis has implications beyond toxicology. The same oxidative and barrier pathways implicated here underlie obesity, diabetes, inflammatory bowel disease, and metabolic syndrome [25]. By defining a common mechanistic axis—oxidative stress, epithelial breach, and microbial imbalance—this review provides a foundation for evaluating how environmental nanoplastics intersect with chronic disease risk. In summary, nanoplastics compromise intestinal homeostasis through linked oxidative, structural, and microbial mechanisms. Recognizing this integrated triad will improve experimental comparability, guide regulatory risk assessment, and inform mitigation strategies aimed at safeguarding long-term human gut health.

## Figures and Tables

**Table 1:** Summary of review articles addressing micro- and nanoplastics (MNPs) and gut-related outcomes. Columns include bibliographic details (Author, Year, Title, Citation), key mechanistic endpoints (epithelial barrier integrity, oxidative stress and ROS, microbiome dysbiosis), scope of species or models covered, and notable remarks. Reviews consistently highlight barrier dysfunction, oxidative stress, and microbial community shifts as recurring consequences of MNP exposure, though emphasis and evidence levels vary across studies. ↑ = increase; ↓ = decrease.

Author	Year	Review Title	Epithelial Barrier Effects	Oxidative Stress/ROS	Microbiome Changes (Dysbiosis)	Species Scope	Notes	Citation
Bora	2024	Microplastics and human health: Unveiling the gut microbiome disruption and chronic disease risks	Weakened barrier integrity, ↑ gut permeability, disruption of tight junctions; systemic translocation of toxins and inflammatory mediators	Discusses oxidative stress, apoptosis, and fibrosis mechanisms in multiple organs (kidney, liver, brain) mediated by dysbiosis	↓ beneficial taxa, ↑ opportunistic/pathogenic taxa; reduced SCFA producers; dysbiosis linked to gut–liver, gut–kidney, gut–brain axes	Human exposure evidence (urine MPs) + rodent models + mechanistic insights	Highlights FMT and probiotics (e.g., *Akkermansia*) as potential interventions; frames MPs as systemic disruptors	[6]
Covello	2024	Micro(nano)plastics and their potential impact on human gut health: A narrative review	↓ tight junction integrity, ↓ mucus secretion, villus/crypt damage, ↑ proinflammatory cytokines; smaller NPs cross epithelium via endocytosis/transcytosis	ROS generation; mitochondrial dysfunction; activation of MAPK, TLR4, NF-κB cascades; apoptosis/necrosis at higher doses	Dysbiosis across *Firmicutes*, *Bacteroidetes*, *Actinobacteria*, *Proteobacteria*; ↓ *Parabacteroides*/SCFA-producers; ↑ *Staphylococcus*, *Lactobacillus*, *Adlercreutzia*, *Bifidobacterium*; inconsistent *Proteobacteria* outcomes	In vitro human cell lines; in vivo mammalian models; notes on aquatic models; human fecal microplastics linked to IBD	Plasticizers and metals may worsen toxicity; microbiota may contribute to plastic biodegradation; human evidence limited/conflicting	[7]
Demarquoy	2024	Microplastics and microbiota: Unraveling the hidden environmental challenge	Editorial mentions barrier dysfunction as a possible consequence of dysbiosis; no primary mechanistic data	Not a central focus; ROS implicated indirectly as part of pollutant–microbiota interactions	Consistently reported ↓ microbial diversity, compositional shifts (*Firmicutes*, *Bacteroidetes*, *Actinobacteria*, *Proteobacteria*, *Verrucomicrobia*) in humans and models	Human observations + environmental/mammalian models (review-level evidence)	Editorial perspective; emphasizes MPs as dysbiosis inducers, possible microbial degradation but most polymers resistant; calls for long-term studies	[8]
Eichinger	2024	Review: Interactions between microplastics and the gastrointestinal microbiome	↓ mucus secretion, tight junction disruption, impaired barrier integrity; translocation of smaller particles into tissues	↑ ROS, mitochondrial dysfunction, oxidative stress linked to inflammation	↑ *Proteobacteria*, *Fusobacteria*, *Firmicutes* (*Staphylococcus*, *Lachnoclostridium*); ↓ *Bacteroides*, *Parabacteroides*, *Akkermansia*, *Bifidobacterium*; dysbiosis linked to inflammation, lipid metabolism changes	Humans, rodents, poultry, fish, ruminants (review synthesis)	Highlights biofilm formation, pollutant and antibiotic resistance gene transfer; some microbial taxa (*Actinobacteria*, *Acinetobacter*) degrade plastics; calls for standardized methods and longitudinal animal studies	[9]
Hirt	2020	Immunotoxicity and intestinal effects of nano- and microplastics: A review of the literature	Reports barrier dysfunction and altered tolerance; immune disruption noted but not fully mechanistic	Mentions oxidative stress as part of immune-inflammatory cascade, but not the central focus	↓ diversity; variable shifts across phyla (↑ *Proteobacteria*, ↓ *Actinobacteria*, ↓ *Firmicutes*); dysbiosis consistently observed	Animal models (mice, zebrafish, others); in vivo studies reviewed	Focus on interplay between microbiota and immunotoxicity; foundational synthesis highlighting host–microbiome imbalance due to MNPs	[10]
Khaledi	2024	The role of gut microbiota in human metabolism and inflammatory diseases: A focus on elderly individuals	Age-related decline in barrier integrity; MNPs may exacerbate permeability and barrier dysfunction in elderly populations	Links dysbiosis to ↑ oxidative stress; MPs/NPs can worsen ROS-related damage in aging gut	↓ diversity, ↓ SCFA-producers (*Faecalibacterium*, *Roseburia*); ↑ opportunistic taxa; MNPs may compound dysbiosis	Human-focused review with emphasis on elderly; references to preclinical and environmental MNP evidence	Therapeutic suggestions include probiotics, prebiotics, dietary modulation, microbiome-targeted interventions	[11]
Kurhaluk	2025	Role of gut microbiota in modulating oxidative stress induced by environmental factors	↓ Tight junction proteins (Claudin, Occludin, ZO-1) and gut vascular barrier disruption (via Wnt/β-catenin) mentioned in pollutant contexts	Emphasis on NF-κB, Nrf2/Keap1, PI3K/Akt, p38-MAPK, JAK/STAT, TLR4/MyD88 as central oxidative stress pathways	Pollutants (toxic metals, nanomaterials, micro/nanoplastics) induce dysbiosis, ↑ inflammation, ↑ resistance gene spread	Broad (rodents, humans, environmental models; review-based)	Identifies antioxidants, probiotics, and prebiotics as potential interventions	[12]
Li	2024	Microplastics in the human body: A comprehensive review of exposure, distribution, migration mechanisms, and toxicity	MPs cross barriers via endocytosis, paracellular leakage, persorption; NPs < 100 nm penetrate tissues; associated with inflammation and fibrosis	ROS generation, mitochondrial dysfunction, apoptosis, immune dysregulation; additives (phthalates, metals) may worsen toxicity	↓ diversity, disruption of SCFA producers, dysbiosis linked to IBD and metabolic diseases	Human-focused review; integrates evidence from clinical findings, in vivo animal models, and in vitro studies	Comprehensive systemic review; highlights biodistribution to intestine, liver, kidney, placenta, brain; calls for standardized exposure models	[13]
Popa	2025	A systematic review of the toxicokinetics of micro- and nanoplastics in mammals following digestive exposure	↓ mucus secretion, villus/crypt damage, barrier dysfunction, ↑ intestinal permeability, inflammation	↑ ROS, hepatic lipid metabolism disruption, oxidative stress implicated in liver, kidney, brain, immune effects	Gut dysbiosis across mammalian models; compositional shifts linked to inflammation and metabolic disruption	Systematic review of 17 in vivo mammalian studies (mice, rats, pigs, guinea pigs)	MP/NP absorption via endocytosis, transcytosis, persorption; systemic distribution to liver, kidney, brain, placenta; chronic/transgenerational effects underexplored	[14]
Souza-Silva	2022	Impact of microplastics on the intestinal microbiota: A systematic review of preclinical evidence	↓ tight junction proteins (ZO-1, Claudin-1), ↓ mucins (Muc1/2/3), ↓ mucus secretion, villi/microvilli damage, ↑ permeability	↑ ROS, CAT, SOD, GstD1, defensins; activation of oxidative stress and immune signaling pathways (TLR2/4, AP-1, IRF5)	↑ *Firmicutes*, *Proteobacteria*, *Verrucomicrobia*, *Chlamydiae*; ↓ *Bacteroidetes*, *Actinobacteria*; ↑ *Lactobacillus*, *Clostridium*, *Vibrio*; ↓ *Bifidobacterium*, *Ruminococcus*	Systematic review of 28 in vivo studies (zeb rafish, mice, worms, insects, crustaceans, mollusks, soil organisms)	Functional changes in microbial metabolism; ↑ antibiotic resistance genes; high risk of bias noted in included studies	[15]
Wang	2024	Microplastic-mediated new mechanism of liver damage: From the perspective of the gut–liver axis	↓ mucus secretion, goblet cell loss, ↑ permeability; barrier disruption allows LPS and MPs to translocate to liver	↑ ROS, hepatic oxidative stress, steatosis, cholestasis, fibrosis; activation of TLR4 pathway	↑ *Proteobacteria*, *Actinobacteria*; ↓ *Bacteroidetes*, *Firmicutes*; dysbiosis linked to liver injury	Animal models (mice, zebrafish, chickens, *tilapia*, *medaka*), liver organoids; limited emerging human data (cirrhosis patients)	Gut–liver axis central to MP toxicity; microbiome-targeted interventions (probiotics, prebiotics, FMT) suggested for mitigation	[16]

## Data Availability

The raw data supporting the conclusions of this article will be made available by the authors upon request.

## Supplementary Materials

The following supporting information can be downloaded at: https://www.mdpi.com/article/10.3390/toxics13121036/s1, Table S1. Search Queries and Sources (Nanoplastics and Microbiome Reviews); Table S2 Summary of Studies with F/B changes; Table S3. Primary in vivo oral-gavage nanoplastic studies (microbiome outcomes, part 2); Table S4. Primary in vivo oral-gavage nanoplastic studies (microbiome outcomes, part 3); Table S5 legend (Gram+/Gram− weighting).

## Abbreviations

The following abbreviations are used in this manuscript:

EPS	exopolysaccharide
F/B	*Firmicutes* and *Bacteroidetes*
GI	gastrointestinal
LPS	lipopolysaccharide
MP	microplastics
MNPs	Micro- and nano-plastics
NP	nanoparticle
PET	polyethylene terephthalate
PP	polypropylene
PE	polyethylene
PS	polystyrene
PS-NPs	Polystyrene nanoplastics
PVC	Polyvinyl chloride
ROS	Reactive Oxygen Species
SCFAs	short-chain fatty acids
SOD	superoxide dismutase
