# Supplementary material for: Role of Nanoplastics in Decreasing the Intestinal Microbiome Ratio: A Review of the Scope of Polystyrene
Source: Toxics. 2025 Nov 29;13(12):1036. doi: 10.3390/toxics13121036 (PMC12737679; doi:10.3390/toxics13121036)
Supplement: Supplementary file 1 [file toxics-13-01036-s001.zip › Table S1.pdf]

Supplemental Materials

The supplemental files consist of this file and four additional *.xlsx files (Tables S2-S5). The titles of Tables S2-S5 are provided following Table S1 for your reference.

Table S1. Search Queries and Sources (Nanoplastics and Microbiome Reviews)

since 2020	**Search Query**	**Source / Platform**	# hits
1	Nanoplastics AND 'Firmicutes/Bacteroidetes ratio' AND dysbiosis AND mice	PubMed	1
2	Polystyrene nanoparticles <500nm AND gut microbiome review	Scopus	0
3	Gut dysbiosis AND plastic nanoparticles AND mammals	Web of Science	9
4	Microplastic nanoparticle effects on intestinal microbial balance	Google Scholar	2
5	Plastic nanoparticles AND Firmicutes AND Bacteroidetes shift	ScienceDirect	4
6	Nanoplastics AND gut microbial community structure	SpringerLink	2

7	Polystyrene NPs AND gut inflammation AND microbiome	Wiley Online Library	3
8	Gut microbiota composition change due to PS-NPs exposure	Taylor & Francis	4
9	F/B ratio disruption AND nano plastic ingestion in rodents	MDPI	1
10	Microbiota alterations from nanoplastic ingestion in zebrafish	Frontiers	2
11	Systematic review of nano-polystyrene AND gut dysbiosis	Elsevier Journals	1
12	Review of plastic nanoparticle toxicity in mammalian gut	Nature Reviews	1
13	Intestinal barrier damage AND nanoplastics AND microbiome review	Oxford Academic	0
14	Impact of polystyrene nanoplastics on gut microbial ecology	SAGE Journals	0
15	Environmental nano plastics AND intestinal microbiota composition	JSTOR	0
16	Nanoplastics AND host-microbiome interactions	BMC	3

17	Plastic nanoparticle ingestion AND F/B ratio trends	Cambridge Core	0
18	Systematic review: nanoparticles AND gut bacterial dysbiosis	BioOne	1
19	Gut microbiome diversity and plastic nanoparticle exposure	PLOS	2
20	Polystyrene nanoplastics and dysbiosis in murine models	Embase	4
21	Gut microbial imbalance due to nanoplastics	bioRxiv	2
22	PS-NPs <500nm ingestion AND intestinal microbiota	medRxiv	0
23	Bacteroidetes abundance AND nano-plastic exposure	DOAJ	1
24	Gut dysbiosis from synthetic nanoparticles ingestion	PubMed	6
25	Review: Plastic NPs AND gut inflammation AND bacteria	Scopus	1
26	Polystyrene nanoplastics ingestion AND microbiome health	Web of Science	3

27	Plastic NPs exposure and intestinal health: review	Google Scholar	2
28	F/B ratio shifts AND synthetic particle ingestion	ScienceDirect	1
29	Nanoplastics AND bacterial diversity loss review	SpringerLink	1
30	Rodent gut dysbiosis AND plastic nanoparticles	Wiley Online Library	4
31	Gut permeability and microbiota under NP stress	Taylor & Francis	2
32	Gut-brain-microbiome axis and polystyrene NPs	MDPI	1
33	Ingestion of NPs and effects on bacterial phyla balance	Frontiers	3
34	Dysbiosis and microbial resilience to plastic NPs	Elsevier Journals	3
35	Reviews on gut microbial disruption by polystyrene NPs	Nature Reviews	1
36	Plastic nanoparticle exposure AND microbial imbalance	Oxford Academic	2
37	Microbiome dysbiosis trends under PS-NP ingestion	SAGE Journals	1

38	Gut flora alteration under polystyrene nanoparticle stress	JSTOR	1
39	Intestinal barrier AND microbiome AND NPs: review	BMC	2
40	Gut microbiota shifts from chronic nanoparticle exposure	Cambridge Core	2
41	Review: microbiome ecology and environmental plastic NPs	BioOne	1
42	Comparative gut microbiome analysis under nano-plastic exposure	PLOS	2
43	Firmicutes suppression AND plastic nanoparticle ingestion	Embase	1
44	Plastic NP exposure AND bacterial metabolic pathways review	bioRxiv	1
Total			84

Table S2 Summary of Studies with F/B changes: **Table S2.xlsx**

Summary of 47 primary studies investigating micro- and nanoplastic (MNP) effects on the gut microbiome across animal and microbial models. Studies encompass a range of polymers (e.g., PS, PP, PE, PET, PVC, PLA, and mixed exposures) and particle sizes, with experimental systems including mice (various C57BL/6 and BALB/c strains, Kunming mice, ICR mice), zebrafish, drosophila, turtles, broiler chickens, and in vitro bacterial or cell culture models (*E. coli, Bacillus, Pseudomonas, Klebsiella*, Caco-2, IEC-6, HepG2). Reported outcomes highlight microbiome composition shifts (46 of 47 studies) such as alterations in Firmicutes/Bacteroidetes ratio, increases in Proteobacteria, or changes in specific genera (e.g., *Lactobacillus*, *Akkermansia*), alongside diversity loss and barrier- or host-relevant effects. Exposure routes included oral gavage, dietary admixture, and waterborne exposures, with doses ranging from environmentally relevant levels to high experimental concentrations. Together, these studies demonstrate consistent microbiome perturbations across taxa and host models, supporting a role for MNPs in disrupting microbial ecology and host–microbiome interactions.

Table S3. Primary in vivo oral-gavage nanoplastic studies (microbiome outcomes, part 2). **Table S3.xlsx**

This table extends the comparative summary begun in Table 2, continuing the study-level charting of in vivo oral nanoplastic exposures. Reported are particle type and size, host/model system, exposure route/duration/dose, and principal microbiome outcomes (F/B ratio, diversity indices, key taxa shifts). The “Notes / other observations” column records unique experimental features, such as weathered or surface-modified particles, waterborne exposures, unusual host systems, or additional host endpoints. Tables 2–4 together provide a continuous dataset covering all included in vivo oral-gavage nanoplastic studies, while Tables S2–S4 organize microbiome outcomes, host endpoints, and study descriptors separately.

Table S4. Primary in vivo oral-gavage nanoplastic studies (microbiome outcomes, part 3). **Table S4.xlsx**

This table continues the comparative summary begun in Tables 2 and 3, presenting study-level details for the remaining in vivo nanoplastic exposures delivered by oral gavage. Reported are particle type and size, host/model system, exposure route/duration/dose, and major microbiome outcomes (F/B ratio, diversity metrics, key taxa shifts). The “Notes / other observations” column highlights unique experimental features (e.g., surface-modified or weathered particles, nonstandard

host models, additional host endpoints, or methodological caveats). Together, Tables 2–4 provide an integrated overview of all included primary studies, while Supplementary Tables S2–S4 separate microbiome outcomes, host endpoints, and study descriptors for detailed reference.

Table S5 legend (Gram+/Gram− weighting). **Table S5.xlsx**
To systematically evaluate how nanoplastic exposure influenced the relative balance of Gram-positive and Gram-negative bacteria, we developed a standardized tallying and weighting strategy. For each study, reported taxa shifts at the phylum or genus level were classified by Gram status, and directional changes were recorded. Gram-positive taxa (e.g., *Firmicutes*, *Lactobacillus*, *Bifidobacterium*, *Actinobacteria*) and Gram-negative taxa (e.g., *Bacteroidetes*, *Proteobacteria*, *Escherichia–Shigella*, *Akkermansia*) were included when authors reported significant increases or decreases.

Each significant increase in a Gram-positive taxon contributed +1 to the “Gram+ ↑ (nw)” column, while each significant decrease contributed +1 to the “Gram+ ↓ (nw)” column. Analogously, increases in Gram-negative taxa contributed +1 to the “Gram− ↑ (nw)” column, and decreases contributed +1 to the “Gram− ↓ (nw)” column. These non-weighted tallies (nw) record every reported change, even if a genus is nested within a phylum.

Weighted tallies (w) were then derived to avoid double-counting nested taxa. For example, if both *Firmicutes* and *Lactobacillus* were reported as decreased, the non-weighted tally counts both (Gram+ ↓ (nw) = 2), but the weighted tally collapses this overlap to one representative decrease (Gram+ ↓ (w) = 1).

A qualitative synthesis of these tallies was then used to assign the overall “Gram+/Gram− (weighted net)” outcome for each study. When decreases in Gram-positive commensals coincided with increases in Gram-negative taxa, the net outcome was recorded as “↓ Gram+/Gram− (dominant Gram− expansion)”. Balanced or nonsignificant effects were classified as “No net effect”, and the reverse pattern (Gram-positive enrichment or Gram-negative loss) was recorded as “↑ Gram+/Gram−.”

Importantly, zeroes and blanks were distinguished to preserve interpretive clarity. Zeroes are data: the authors analyzed and explicitly reported taxa but found none in that category (e.g., 0 Gram− increases). Blanks are missing: the authors did not measure or report outcomes for that category (e.g., no microbiome sequencing performed, or no F/B ratio reported).

This structured approach ensured consistency across heterogeneous studies and allowed for direct comparison of Gram-positive versus Gram-negative bacterial responses to nanoplastic exposure.
